# Adrenal-Permissive Germline *HSD3B1* Allele and Prostate Cancer Outcomes

**DOI:** 10.1001/jamanetworkopen.2024.2976

**Published:** 2024-03-20

**Authors:** Rana R. McKay, Tyler J. Nelson, Meghana S. Pagadala, Craig C. Teerlink, Anthony Gao, Alex K. Bryant, Fatai Y. Agiri, Kripa Guram, Reid F. Thompson, Kathryn M. Pridgen, Tyler M. Seibert, Kyung Min Lee, Hannah Carter, Julie A. Lynch, Richard L. Hauger, Brent S. Rose

**Affiliations:** 1 Division of Hematology-Oncology, Department of Internal Medicine, University of California, San Diego, La Jolla; 2 Veterans Affairs Informatics and Computing Infrastructure (VINCI), Veterans Affairs Salt Lake City Health Care System, Salt Lake City, Utah; 3 Department of Radiation Medicine and Applied Sciences, University of California San Diego, La Jolla; 4 Veterans Affairs San Diego Healthcare System, San Diego, California; 5 Division of Epidemiology, Department of Internal Medicine, University of Utah School of Medicine, Salt Lake City; 6 Department of Radiation Oncology, University of Michigan, Ann Arbor; 7 Department of Radiation Oncology, Veterans Affairs Ann Arbor Health System, Ann Arbor, Michigan; 8 Department of Radiation Medicine, Oregon Health and Sciences University, Portland; 9 Division of Hospital and Specialty Medicine, Veterans Affairs Portland Healthcare System, Portland, Oregon; 10 Department of Bioengineering, University of California, San Diego, La Jolla; 11 Department of Radiology, University of California, San Diego, La Jolla; 12 Division of Medical Genetics, Department of Medicine, University of California, San Diego, La Jolla; 13 Center for Behavioral Genetics of Aging, University of California San Diego, La Jolla

## Abstract

**Importance:**

The adrenal androgen–metabolizing 3β-hydroxysteroid dehydrogenase-1 enzyme, encoded by the *HSD3B1* gene, catalyzes the rate-limiting step necessary for synthesizing nontesticular testosterone and dihydrotestosterone production. The common adrenal-permissive *HSD3B1*(1245C) allele is responsible for encoding the 3β-HSD1 protein with decreased susceptibility to degradation resulting in higher extragonadal androgen synthesis. Retrospective studies have suggested an association of the *HSD3B1* adrenal-permissive homozygous genotype with androgen deprivation therapy resistance in prostate cancer.

**Objective:**

To evaluate differences in mortality outcomes by *HSD3B1* genetic status among men with prostate cancer.

**Design, Setting, and Participants:**

This cohort study of patients with prostate cancer who were enrolled in the Million Veteran Program within the Veterans Health Administration (VHA) system between 2011 and 2023 collected genotyping and phenotyping information.

**Exposure:**

*HSD3B1* genotype status was categorized as AA (homozygous adrenal-restrictive), AC (heterozygous adrenal-restrictive), or CC (homozygous adrenal-permissive).

**Main Outcomes and Measures:**

The primary outcome of this study was prostate cancer–specific mortality (PCSM), defined as the time from diagnosis to death from prostate cancer, censored at the date of last VHA follow-up. Secondary outcomes included incidence of metastases and PCSM in predefined subgroups.

**Results:**

Of the 5287 participants (median [IQR] age, 69 [64-74] years), 402 (7.6%) had the CC genotype, 1970 (37.3%) had the AC genotype, and 2915 (55.1%) had the AA genotype. Overall, the primary cause of death for 91 patients (1.7%) was prostate cancer. Cumulative incidence of PCSM at 5 years after prostate cancer diagnosis was higher among men with the CC genotype (4.0%; 95% CI, 1.7%-6.2%) compared with the AC genotype (2.1%; 95% CI, 1.3%-2.8%) and AA genotype (1.9%; 95% CI, 1.3%-2.4%) (*P* = .02). In the 619 patients who developed metastatic disease at any time, the cumulative incidence of PCSM at 5 years was higher among patients with the CC genotype (36.0%; 95% CI, 16.7%-50.8%) compared with the AC genotype (17.9%; 95% CI, 10.5%-24.7%) and AA genotype (18.5%; 95% CI, 12.0%-24.6%) (*P* = .01).

**Conclusions and Relevance:**

In this cohort study of US veterans undergoing treatment for prostate cancer at the VHA, the *HSD3B1* CC genotype was associated with inferior outcomes. The *HSD3B1* biomarker may help identify patients who may benefit from therapeutic targeting of 3β-hydroxysteroid dehydrogenase-1 and the androgen-signaling axis.

## Key Points

**Question:**

Is the adrenal-permissive *HSD3B1* homozygous genotype associated with worse clinical outcomes in men with prostate cancer?

**Findings:**

In this cohort study of 5287 men with prostate cancer in the Million Veteran Program, the *HSD3B1* adrenal-permissive homozygous genotype (compared with the adrenal-restrictive homozygous and heterozygous genotype) was associated with worse prostate cancer–specific mortality. Subset analysis of metastatic prostate cancer also showed worse prostate cancer–specific mortality in the adrenal-permissive homozygous genotype group.

**Meaning:**

These findings suggest that the *HSD3B1* adrenal-permissive homozygous genotype is associated with inferior outcomes in men with prostate cancer.

## Teaser

This cohort study analyzes the association of *HSD3B1* status with prostate cancer outcomes among patients in the Veterans Affairs Health System in the US.

## Introduction

The longstanding frontline treatment for patients with prostate cancer has been androgen deprivation therapy (ADT) through either surgical or medical castration. Castration results in decreased circulating and intratumoral androgen production, preventing prostate cancer progression. Prostate cancer pathogenesis is dependent on oncogenic activation of androgen receptor (AR) signaling by testosterone and the more potent 5α-reduced metabolite, 5α-dihydrotestosterone (DHT).^1^ Although patients are usually responsive to ADT initially, a subset of patients develop castration-resistant prostate cancer (CRPC) with poor prognosis.

Multiple androgen-dependent mechanisms of resistance to ADT have been characterized, including AR overexpression, acquisition of constitutively active AR slice variants, gain-of-function AR variants, dysregulated AR coactivators and corepressors that sensitize AR to ligand binding, extragonadal androgen synthesis from adrenal precursor steroids, and de novo synthesis from cholesterol by the enzyme 3β-hydroxysteroid dehydrogenase-1 (3β-HSD1).^1^ The 3β-HSD1 enzyme, encoded by the *HSD3B1* gene, catalyzes the rate-limiting step in the conversion of dehydroepiandrosterone (DHEA) to androstenedione and androstenedione to testosterone and DHT, generating a nontesticular source of testosterone and DHT.^2,3^ *HSD3B1* has a common single-nucleotide variant where 2 different germline, missense-encoding alleles result in distinct functional activities of the 3β-HSD1 protein.^4^ *HSD3B1*(1245A) is the adrenal-restrictive allele, encoding for a more rapidly degraded enzyme that restricts conversion of DHEA to testosterone and DHT.^5^ *HSD3B1*(1245C) is the adrenal-permissive allele that encodes for a stable enzyme, resistant to ubiquitination and proteosome degradation, resulting in sustained 3β-HSD1 levels and more robust DHEA-sulfate conversion to testosterone and DHT, thereby generating higher downstream exposure of the prostate to potent androgens.^4^

Growing evidence demonstrates an association of *HSD3B1* inheritance with prostate cancer outcomes. Several independent cohorts have identified that the adrenal-permissive *HSD3B1*(1245C) allele is associated with worse outcomes in patients postprostatectomy^6^ and postradiotherapy.^7^ Additionally, data suggests inferior outcomes in the setting of metastatic, hormone-sensitive prostate cancer^8^ and CRPC.^9^ Several studies^7,9,10^ have highlighted that the presence of the adrenal-permissive *HSD3B1*(1245C) allele is associated with resistance to ADT, AR signaling agents, and CYP17A1 inhibition. The outcome is largely associated with patients who are homozygous for *HSD3B1*(1245C) with more variable outcomes for heterozygous patients.^11^

Across existing studies with sample sizes ranging from 102 to 546 patients, approximately 36% to 42% of patients were heterozygous for the adrenal-permissive *HSD3B1*(1245C) allele and 6% to 9% were homozygous for the *HSD3B1*(1245C) allele.^6,7,8,9,10,12,13,14^ The population frequency of the adrenal-permissive *HSD3B1*(1245C) allele varies widely by ancestry (34% European, 20% American, 16% South Asian, 9% African, and 8% East Asian).^6^ Although the *HSD3B1*(1245C) allele frequency is highest among White men, existing studies have included a very limited number of Black patients. Most established studies did not report on race and of 2 studies that reported on race, 12 of 118 patients (10.2%) in Hearn et al^6^ (8 homozygous adrenal-restrictive patients, 4 heterozygous patients, and 0 homozygous patients) and 27 of 246 patients (11.0%) in Lu et al^9^ (27 adrenal-restrictive homozygous or heterozygous patients and 0 homozygous patients) were Black individuals.^6,9^

The objective of this study was to evaluate the differences in outcomes in men with prostate cancer based on *HSD3B1* genetic status. We hypothesized that patients who were homozygous for the adrenal-permissive *HSD3B1*(1245C) allele would have worse prostate cancer–related survival outcomes. Given the prognostic and potential therapeutic implications of *HSD3B1* genetic status on outcomes, we used the large, ethnically diverse Million Veteran Program (MVP) data set to better inform our understanding of *HSD3B1* genetic status in patients with prostate cancer.

## Methods

### Data Source

This retrospective cohort study used data analyzed as part of an MVP research study protocol that was approved by the US Department of Veterans Affairs (VA) central institutional review board, as well as the research and development committees at the San Diego, California VA and Salt Lake City, Utah VA. All participants provided written informed consent and Health Insurance Portability and Accountability Act authorization. This study followed the Strengthening the Reporting of Observational Studies in Epidemiology (STROBE) reporting guideline.

Data on clinical characteristics and demographics for veteran men with prostate cancer were extracted from the Veterans Healthcare Administration (VHA) Corporate Data Warehouse, which includes a cancer registry of patients diagnosed and/or treated for any cancer at any VHA site and specific data products to facilitate research. We used the VA Informatics and Computing Infrastructure Prostate Cancer Data Core to ascertain certain clinical outcomes. Ascertainment of metastases in the Prostate Cancer Data Core was accomplished using a natural language processing algorithm to scan clinical notes and has been previously described elsewhere.^15^ Cause of death was ascertained through the National Death Index. Patients with a primary cause of death code (C61) were counted as having a prostate cancer–related death for the end point of prostate cancer–specific mortality (PCSM). We used genetic data from the MVP genetic repository.

### Study Population

The MVP includes approximately 650 000 individuals. Using the previously described natural language processing methods,^15^ we extracted data for men who developed incident prostate cancer in the VHA between 2011 and 2023. We limited our cohort to patients diagnosed with prostate cancer after their enrollment in MVP (9291 patients). Patients with missing genotype and clinical information were excluded. Missing demographic information (ie, unknown race and smoking status) is reported in Table 1. Race was self-reported; however, in the MVP database, self-reported Black race is greater than 99% concordant with African ancestry. Participants selected from the following options for race: American Indian or Alaska Native, Asian, Black, Native Hawaiian or Other Pacific Islander, White, and multiple races. Race was assessed to enable investigation of race-based differences by *HSD3B1* allele status.

**Table 1.** Baseline Clinical and Disease Characteristics

Characteristic	Participants, No. (%) (N =5287)			*P* value
	*HSD3B1* AA (n =2915)	*HSD3B1* AC (n = 1970)	*HSD3B1* CC (n = 402)	
Self-reported race				
American Indian or Alaska Native	20 (0.7)	13 (0.7)	<11 (<2.7)	<.001
Asian	19 (0.7)	<11 (<0.6)	0	
Black	1209 (41.5)	339 (17.2)	19 (4.7)	
Multiracial	<11 (<0.4)	<11 (<0.6)	0	
Native Hawaiian or Other Pacific Islander	22 (0.8)	16 (0.8)	<11 (<2.7)	
White	1498 (51.4)	1520 (77.2)	369 (91.8)	
Unknown	138 (4.7)	80 (4.1)	12 (3.0)	
Age, y				
≤60	435 (14.9)	206 (10.5)	29 (7.2)	<.001
61-70	1418 (48.6)	879 (44.6)	182 (45.3)	
>70	1062 (36.4)	885 (44.9)	191 (47.5)	
Smoking history				
None	775 (26.6)	547 (27.8)	119 (29.6)	.001
Ever	1547 (53.1)	951 (48.3)	182 (45.3)	
Unknown	593 (20.3)	472 (24.0)	101 (25.1)	
Gleason category				
≤6	806 (27.7)	551 (28.0)	95 (23.6)	.35
7	1375 (47.2)	929 (47.2)	206 (51.2)	
8	355 (12.2)	225 (11.4)	40 (10.0)	
≥9	379 (13.0)	265 (13.5)	61 (15.2)	
Prostate specific antigen at diagnosis, median (IQR), ng/mL^a^	4.80 (0.75-7.96)	4.58 (0.66-7.70)	4.22 (0.50-7.91)	.23
Metastases at diagnosis	219 (7.5)	150 (7.6)	33 (8.2)	.89
Treatment				
Any radiation therapy	1663 (57.0)	1110 (56.3)	214 (53.2)	.35
Prostatectomy	588 (20.2)	415 (21.1)	86 (21.4)	.69
Androgen deprivation therapy within 6 mo of diagnosis	632 (21.7)	425 (21.6)	87 (21.6)	>.99

Abbreviations: AA, adrenal-restrictive homozygous; AC, adrenal-restrictive heterozygous; CC, adrenal-permissive homozygous.

^a^ To convert to micrograms per liter, multiply by 1.

### *HSD3B1* Genotyping

All study participants provided blood samples for DNA extraction and genotyping. Blood samples were collected and banked at the VA Central Biorepository in Boston, Massachusetts. DNA extracted from buffy coat was genotyped using a custom affymetrix axiom biobank array of 723 305 variants.^16^ While the details on the quality control and imputation have been described previously,^17^ briefly, the imputation process used approximately 100 000 multiethnic background, whole genomes available from TopMed and the *HSD3B1* allele was imputed with INFO-R2 = 0.98061. *HSD3B1* genotype status, which was imputed, was categorized as an ordinal variable with patients classified as adrenal-restrictive homozygous (AA), adrenal-restrictive heterozygous (AC), and adrenal-permissive homozygous (CC).

### Study Design and End Points

The primary outcome of this study was PCSM, defined as the time from diagnosis to death from prostate cancer, censored at the date of last VA follow-up or date known to be alive in the VHA medical record. Secondary outcomes included incidence of metastases, defined as the time from diagnosis to metastasis development, censored at the date of last VA follow-up or date known to be alive in the VHA medical record. PCSM was evaluated by *HSD3B1* genotype status in the overall cohort and in patients with metastatic disease.

### Bias Prevention

We limited our cohort to patients diagnosed with prostate cancer after their enrollment in MVP to avoid survival bias and prevent an overrepresentation of individuals with the AA or AC genotype. Given concern about accuracy of timing of metastatic CRPC (mCRPC) diagnosis date, we did not conduct an analysis looking at rates and time to mCRPC.

### Statistical Analysis

We compared distributions of baseline clinical and demographic information using 1-way analysis of variance tests (which compare the means of 2 or more groups to determine if they are significantly different from each other) for continuous variables and χ^2^ tests for categorical variables to determine if distributions across categories were the same between 2 sets of data. We used the CreateTableOne function in R statistical software version 4.0.3 with the package tableone (R Project for Statistical Computing). Cumulative incidence functions of PCSM were calculated and plotted using the cuminc function in R with the package cmprsk. Cumulative incidences measure the combined number events over a time period while accounting for censoring; they are compared using the Gray test. We used multivariable Cox proportional hazards regressions, which measure associations of 1 or more factors and risk of an event for time to event data, adjusting for *HSD3B1* status, age, race, Gleason score, prostate-specific antigen (PSA) level at diagnosis, cancer stage at diagnosis, and treatment to measure associations of *HSD3B1* genotypes with outcomes; proportional hazards assumptions were verified using the coxph function in R with the package survival. We created models using 2 different index dates: either date of diagnosis of prostate cancer or date of development of metastases (Table 2). Hazard ratios (HRs) are reported with their 95% CIs and a 2-sided *P* < .05 was considered significant.

**Table 2.** Multivariable Cox Regression Analysis Evaluating Variables Associated With Prostate Cancer–Specific Mortality From Time of Diagnosis, Time to Metastasis Development in Patients With Nonmetastatic Disease, and Time From Metastases Development to Death From Prostate Cancer

Variable	Total cohort PCSM (time from diagnosis to prostate cancer death) (n = 5287; events = 91)		Time from diagnosis to metastasis (n = 4885; events = 217)		Time from metastases to prostate cancer death (n = 619; events = 74)	
	HR (95% CI)	*P* value	HR (95% CI)	*P* value	HR (95% CI)	*P* value
*HSD3B1* genotype						
AA and AC	1 [Reference]	NA	1 [Reference]	NA	1 [Reference]	NA
CC	2.14 (1.20-3.83)	.01	1.02 (0.63-1.67)	.92	2.48 (1.34-4.58)	.004
Self-reported race						
Non-Black^a^	1 [Reference]	NA	1 [Reference]	NA	1 [Reference]	NA
Black^b^	0.77 (0.44-1.35)	.36	0.74 (0.53-1.03)	.07	0.94 (0.50-1.76)	.85
Age (per 5 y)	1.28 (1.08-1.50)	.003	1.18 (1.06-1.32)	.002	1.23 (1.03-1.47)	.02
Gleason score						
≤7	1 [Reference]	NA	1 [Reference]	NA	1 [Reference]	NA
8	3.06 (1.54-6.05)	.001	2.23 (1.50-3.30)	<.001	3.19 (1.35-7.51)	.008
9-10	4.96 (2.66-9.23)	<.001	4.88 (3.54-6.74)	<.001	4.44 (2.02-9.75)	<.001
Prostate specific antigen at diagnosis	1.001 (1.000-1.001)	<.001	1.015 (1.010-1.020)	<.001	1.001 (1.000-1.001)	<.001
Stage at diagnosis						
Nonmetastatic	1 [Reference]	NA	1 [Reference]	NA	1 [Reference]	NA
Metastatic	11.73 (7.04-19.54)	<.001	NA	NA	NA	NA
Treatment						
Radiation therapy						
None	1 [Reference]	NA	1 [Reference]	NA	1 [Reference]	NA
Any	1.16 (0.74-1.81)	.51	0.95 (0.71-1.26)	.72	1.24 (0.76-2.05)	.39
Androgen deprivation therapy within 6 mo of diagnosis vs no ADT within 6 mo of diagnosis						
No	1 [Reference]	NA	1 [Reference]	NA	1 [Reference]	NA
Yes	1.37 (0.85-2.21)	.20	1.1 (0.79-1.53)	.58	1.16 (0.70-1.93)	.56

Abbreviations: AA, adrenal-restrictive homozygous; AC, adrenal-restrictive heterozygous; CC, adrenal-permissive homozygous; HR, hazard ratio; NA, not applicable; PCSM, prostate cancer specific mortality.

^a^ Non-Black included American Indian or Alaska Native, Asian, Native Hawaiian or Other Pacific Islander, White, multiple races, and unknown.

^b^ Black race included as a variable because there is a well-described disparity in outcomes among Black patients that has not been seen across other race categories.

## Results

### Baseline Characteristics

We identified 9291 men with incident prostate cancer after enrollment in the MVP. We excluded 3807 individuals with unknown Gleason scores and 197 individuals with unknown PSA at diagnosis for a final cohort of 5287 patients (median [IQR] age, 69 [64-74] years) (eFigure in Supplement 1). Of the 5287 patients, 1567 participants (29.6%) self-identified as Black, and 3387 (64.1%) self-identified as White. Race categories containing fewer than 11 participants are reported as less than 11 to ensure that patient data remains properly deidentified (Table 1). Among the entire cohort, 402 men (7.6%) had the CC genotype, 1970 men (37.3%) had the AC genotype, and 2915 men (55.1%) had the AA genotype. Among self-identified Black individuals, 19 (1.2%) had the CC genotype, 339 (21.6%) had the AC genotype, and 1209 (77.2%) had the AA genotype. Among self-identified White individuals, 369 (10.9%) had the CC genotype, 1520 (44.9%) had the AC genotype, and 1498 (44.2%) had the AA genotype. Black men were more prominently represented in the AA genotype group than in the AC and CC groups, comprising 41.5% of the AA group (1209 of 2915 participants), 17.2% of the AC group (339 of 1970 participants), and 4.7% of the CC group (19 of 402 participants). Gleason grade, PSA at time of diagnosis, and rates of metastases at diagnoses (AA, 219 of 2915 participants [7.5%]; AC, 150 of 1970 participants [7.6%]; CC, 33 of 402 participants [8.2%]) were not significantly different between genotype groups. Of the 5287 patients, 2987 (56.5%) received radiation therapy while 1089 (20.6%) received prostatectomy. There were no significant differences in the rates of treatments by genotype (Table 1).

### Survival Analysis

Over the course of the study period, prostate cancer was the primary cause of death for 91 patients (1.7%). Prostate cancer deaths were significantly higher in patients with the *HSD3B1* CC genotype (14 of 402 patients [3.5%]) compared with the *HSD3B1* AC (33 of 1970 patients [1.7%]) and AA genotypes (44 of 2915 participants [1.5%]). Of the 5287 patients, 619 (11.7%) developed metastases, and the percentage of patients with metastases at any point was similar between the AA (335 of 2915 patients [11.5%]), AC (232 of 1970 patients [11.8%]), and CC (51 of 402 patients [12.7%]) genotype groups. Metastases within 1 year of diagnosis were found in 219 patients with the AA genotype (7.5%), 150 patients with the AC genotype (7.6%), and 33 patients with the CC genotype (8.2%). A total of 312 patients (5.9%) died from other causes. Non–prostate cancer mortality was similar in all *HSD3B1* groups with 171 events in patients with the AA genotype (5.9%), 116 events (5.9%) in patients with the AC genotype, and 25 events (6.2%) in patients with the CC genotype.

**Figure 1.**
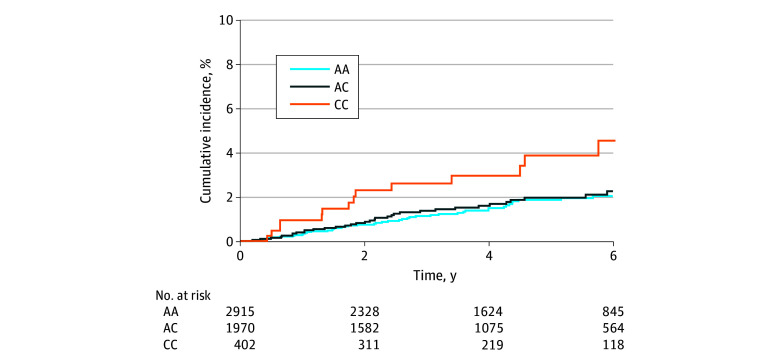
Cumulative Incidence of Prostate Cancer–Specific Mortality Within *HSD3B1* Groups AA indicates the adrenal-restrictive homozygous genotype; AC, the adrenal-restrictive heterozygous genotype; and CC, the adrenal-permissive homozygous genotype.

**Figure 2.**
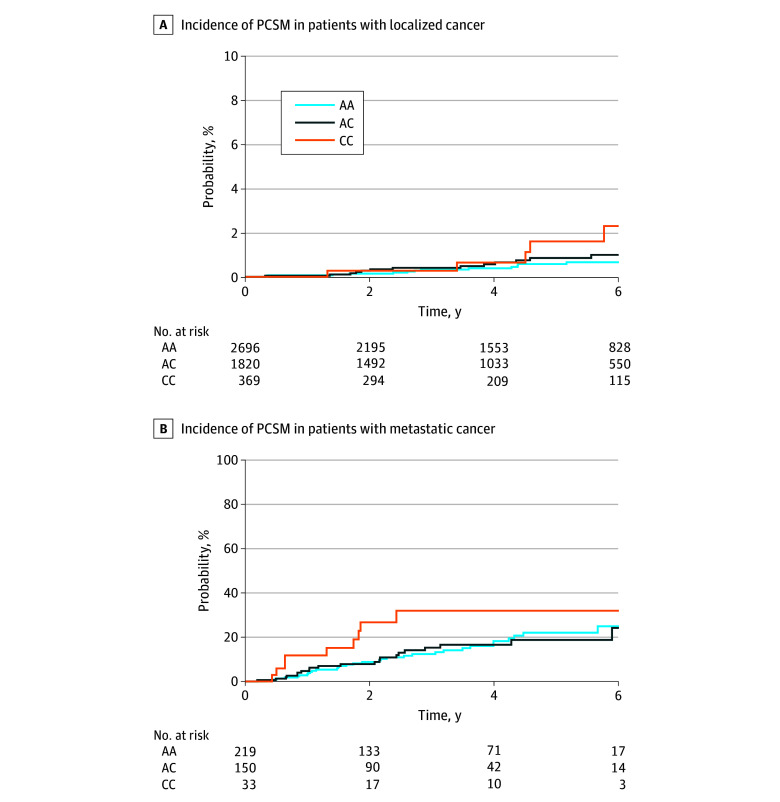
Cumulative Incidence of Prostate Cancer–Specific Mortality Among Patients With and Without Metastatic Prostate Cancer The figure shows the cumulative incidence of prostate cancer–specific mortality among patients with nonmetastatic prostate cancer at diagnosis (A) and the cumulative incidence of prostate cancer–specific mortality from date of prostate cancer diagnosis among patients with metastatic prostate cancer within 1 year of prostate cancer diagnosis (B). AA indicates the adrenal-restrictive homozygous genotype; AC, the adrenal-restrictive heterozygous genotype; and CC, the adrenal-permissive homozygous genotype.

**Figure 3.**
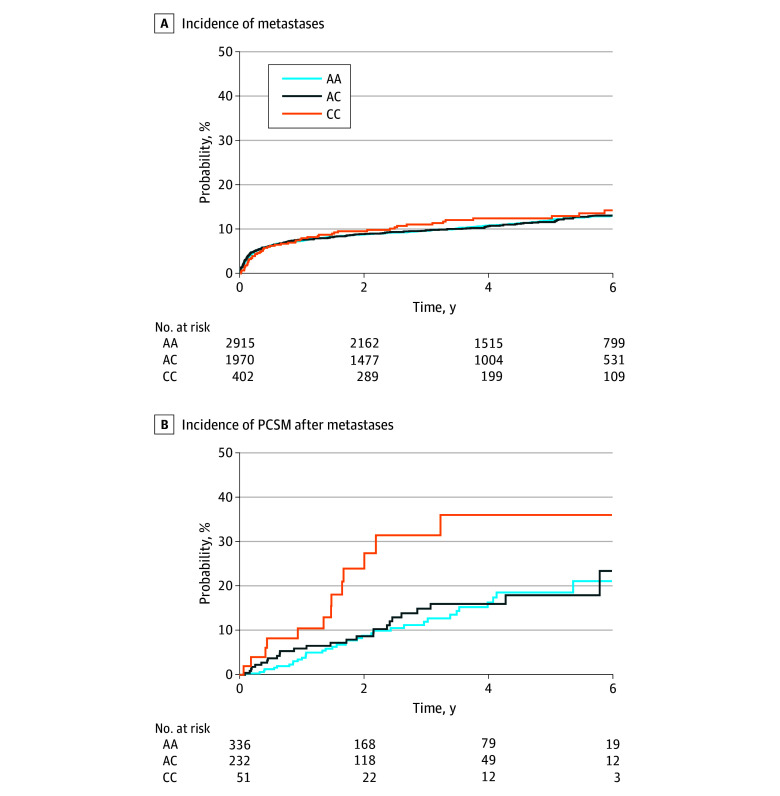
Cumulative Incidence of Development of Metastases and Prostate Cancer–Specific Mortality Among Patients With Metastatic Prostate Cancer The figure shows the cumulative incidence of development of metastases in patients with nonmetastatic prostate cancer at diagnosis (A) and the cumulative incidence of prostate cancer–specific mortality from time of development of metastases among patients who developed metastases (B). AA indicates the adrenal-restrictive homozygous genotype; AC, the heterozygous genotype; and CC, the adrenal-permissive homozygous genotype.

Median (IQR) follow-up in the cohort was 4.4 (2.4-6.5) years. The cumulative incidence of PCSM at 5 years after diagnosis of prostate cancer was higher among patients with the *HSD3B1* CC genotype (4.0%; 95% CI, 1.7%-6.2%) compared with the AC genotype (2.1%; 95% CI, 1.3%-2.8%) and AA genotype (1.9%; 95% CI, 1.3%-2.4%) (*P* = .02) (Figure 1). In the subset of patients who were diagnosed with nonmetastatic prostate cancer at diagnosis, cumulative incidence of PCSM was higher in individuals with CC genotypes (1.8%; 95% CI, 0.0%-3.5%) compared with individuals with AA genotypes (0.6%; 95% CI, 0.2%-0.9%) or AC genotypes (0.9%; 95% CI, 0.4%-1.5%) (*P* = .04). Cumulative incidence of PCSM was similar among genotype groups in individuals with metastatic disease at diagnosis (AA, 21.7% [95% CI, 13.8%-28.9%]; AC, 19.0% [95% CI, 10.3%-26.9%]; CC, 29.9% [95% CI, 9.3%-45.8%]; *P* = .28) (Figure 2). The cumulative incidence of metastases at 5 years from diagnosis was similar between *HSD3B1* genotype groups (AA, 12.3% [95% CI, 10.9%-13.6%]; AC, 11.8% [95% CI, 10.2%-13.4%]; CC, 13.2% [95% CI, 9.6%-16.6%]; *P* = .79) (Figure 3). In patients who developed metastatic disease at any time, the cumulative incidence of PCSM at 5 years after development of metastases was higher in the *HSD3B1* CC genotype group (36.0%; 95% CI, 16.7%-50.8%) compared with the AC genotype group (17.9%; 95% CI, 10.5%-24.7%) and AA genotype group (18.5%; 95% CI, 12.0%-24.6%) (*P* = .01) (Figure 3).

### Multivariable Analysis

We observed higher PCSM in men with the *HSD3B1* CC genotype compared with men with the AC or AA genotype (HR, 2.14; 95% CI, 1.20-3.83; *P* = .01) (Table 2). We found no association of the *HSD3B1* CC genotype with development of metastases (HR, 1.02; 95% CI, 0.63-1.67, *P* = .92); however, in patients who developed metastatic disease at any time, the *HSD3B1* CC genotype was associated with worse PCSM from time of metastases (HR, 2.48; 95% CI, 1.34-4.58; *P* = .004). Other variables associated with PCSM included age at diagnosis, Gleason score at diagnosis, PSA at diagnosis, and presence of metastases at diagnosis. These variables were also associated with shorter time to metastases and time from metastases to death. In this cohort, Black race was not associated with worse PCSM from time of diagnosis, time from diagnosis to metastases, or time from metastases to death from prostate cancer. Subset analysis in Black men could not be performed due to small sample size.

## Discussion

In this cohort study, we investigated the association of *HSD3B1* genotype status with outcomes in a large cohort of men with prostate cancer who were receiving care in the VA health care system and underwent germline genotyping in the MVP. Our findings suggest the *HSD3B1* CC genotype is associated with inferior PCSM among all patients and in individuals who develop metastatic disease. Additionally, we found that the *HSD3B1* CC genotype is less common in Black men than in White men. To our knowledge, this is the largest study to date investigating the prognostic significance of *HSD3B1* genotype status in prostate cancer within a racially heterogeneous patient population.

We have demonstrated that men who are homozygous for the *HSD3B1* adrenal-permissive CC genotype have worse PCSM compared with those with adrenal-restrictive homozygous (AA) or heterozygous (AC) genotypes. Our findings are consistent with other studies that have investigated the prognostic significance of *HSD3B1* in prostate cancer.^6,7,12^ Hearn and colleagues^6^ were early investigators of patient outcomes by *HSD3B1* genotype status in men who had received treatment with ADT.^18^ Those with the *HSD3B1* CC genotype demonstrated worse progression-free survival, distant metastasis-free survival, and overall survival as a function of the number of adrenal-permissive alleles inherited in a primary cohort of 443 patients postprostatectomy and in 2 validation cohorts (post-prostatectomy and metastatic).

The function of *HSD3B1* is critically relevant in the setting of androgen deprivation. In the absence of ADT, androgen supply is dominated by testicular synthesis and secretion of testosterone and DHT. With pituitary-gonadal axis suppression by ADT, circulating levels of testosterone and DHT decline dramatically; however, tumor growth can persist through persistent exposure of the prostate to intratumoral androgens derived from either adrenocortical precursor steroids or de novo synthesis from cholesterol.^18^ In 2013, Chang and colleagues^4^ identified that a minor allele in *HSD3B1* conferred a novel resistance mechanism to ADT. 3β-HSD1 catalyzes the irreversible and rate-limiting step of DHEA conversion to androstenedione.^4^ DHEA itself has no androgenic effect given its low affinity for the AR.^19^ However, the adrenal-permissive allele *HSD3B1*(1245C) encodes for a stable enzyme, resistant to ubiquitination and proteosome degradation, resulting in more robust DHEA-sulfate conversion to the more potent androgens (testosterone and DHT).^4^

To investigate the prognostic significance of *HSD3B1* among individuals likely to be receiving ADT, we evaluated outcomes by *HSD3B1* status in a cohort of 619 men with prostate cancer who developed metastases. In this subgroup, men with the *HSD3B1* CC genotype had significantly worse PCSM compared with men with the AC or AA genotype. Interestingly, the prognostic significance of the *HSD3B1* adrenal-permissive genotype is relevant to other hormonally driven tumors, including estrogen receptor-positive breast cancer, human epidermal growth factor receptor–negative breast cancer, and endometrial cancer, which may potentially be associated with promoting androstenedione conversion to estrone by aromatase, thereby contributing to worse clinical outcomes.^20^

Subsequent studies^10^ have evaluated the prognostic role of the *HSD3B1* genotype CC in patients receiving abiraterone or enzalutamide in CRPC. One of the largest studies by Khalaf et al^10^ included 547 men with mCRPC treated with abiraterone or enzalutamide, of whom 15% harbored the *HSD3B1* CC genotype. The study included a predominately docetaxel-naive cohort (cohort 1) and a docetaxel-pretreated cohort (cohort 2). Compared with patients with the *HSD3B1* AA or CC genotype, patients with the CC genotype demonstrated lower PSA response rates and shorter time to progression with abiraterone or enzalutamide in both cohorts; however, shorter overall survival was observed only in the docetaxel-naive cohort.^10^ Lu et al^9^ evaluated the outcomes of 266 patients with mCRPC treated with abiraterone or enzalutamide, of whom 8.3% had the *HSD3B1* CC genotype. While they did not demonstrate a difference in PSA outcomes and treatment duration with abiraterone or enzalutamide, overall survival was shorter in patients with the CC genotype.^9^ These 2 studies^9,10^along with other published studies relay a consistent message that clinical outcomes are worse among individuals with the *HSD3B1* CC genotype.

Several studies^21,22,23^ have investigated the mechanistic underpinning behind *HSD3B1* resistance. Because abiraterone is metabolized by 3β-HSD1, the *HSD3B1* genetic switch can increase or reduce the degradation and metabolism of abiraterone, thereby regulating its therapeutic effect.^23^ Furthermore, Mei and colleagues^23^ demonstrated that 3β-HSD1 impairs enzalutamide action through enhanced steroidogenesis of potent androgens in addition to promoting metabolism of abiraterone and reducing drug concentration and effectiveness. In their study,^23^ genetically augmented 3β-HSD1 activity upregulated accumulation of intratumor DHT that possesses a substantially higher affinity for AR compared with the weaker affinity of enzalutamide. The competitive kinetics favor DHT preferentially binding to AR over enzalutamide, diminishing its antagonism of AR. Given the implications of *HSD3B1* in promoting resistance, there is rationale to support therapeutic targeting through 3β-HSD1 inhibition and other therapies targeting the androgen-receptor axis.

Our study underscores the race-related differences in *HSD3B1* genetics. Our cohort included a large population of Black men genotyped for *HSD3B1* (1567 patients). Only 19 Black patients (1.2%) had the *HSD3B1* CC genotype, compared with 383 White patients (10.3%). The small number of Black patients precluded subset analyses by race. Several studies^24,25^ suggest that abiraterone is associated with improved prostate cancer outcomes among Black patients compared with non-Hispanic White men. It is plausible to hypothesize that differences in *HSD3B1* among racial groups could partially explain these observations, although further studies are warranted.

Our multivariable analysis demonstrated a significant association of *HSD3B1* genotype with PCSM. Known prognostic factors including Gleason score, PSA at diagnosis, and stage at diagnosis were also associated with PCSM. We also demonstrated that increasing age was associated with worse outcomes. Consistent with other analyses from the VA health care system^26,27^ (where access to care is more consistent), Black race was not associated with worse outcomes in our study. Future research tracking PSA before and after ADT to assess response based on *HSD3B1* genotype are needed.

### Limitations

Although this is, to our knowledge, the largest, most racially diverse cohort to date investigating the association of the *HSD3B1* genotype in men with prostate cancer, there are several limitations. All patients included in the database were veterans of the US military, which could decrease study generalizability. Although our study included a large cohort of Black men, the proportion of Black patients with the *HSD3B1* CC genotype was small, precluding subset analyses by race. We attempted to capture prostate cancer–related deaths with the end point of PCSM, which may be subject to cause of death misattributions. In addition, we could not consistently capture use and duration of ADT or other androgen receptor signaling inhibitors, including abiraterone, enzalutamide, apalutamide, and darolutamide. We did not investigate PSA changes following ADT by *HSD3B1* genotype status. Our analysis only analyzed inherited germline variants in *HSD3B1* and did not investigate somatically acquired variants.

## Conclusions

In this cohort study of US veterans undergoing treatment for prostate cancer in the VA health care system who had undergone germline genotyping, we investigated the association of *HSD3B1* genotype status with outcomes. We found that patients homozygous for the *HSD3B1* adrenal-permissive CC genotype had worse PCSM. Additionally, our findings highlight the higher prevalence of the AA and AC genotype among Black men as compared with White men. Our study adds to growing evidence of the prognostic importance of *HSD3B1* and provides further support for therapeutic targeting of this pathway.

## Notes

Supplement 1. **eFigure.** Cohort Selection

Supplement 2.
Data Sharing Statement
