# Supplementary material for: Adrenal-Permissive Germline HSD3B1 Allele and Prostate Cancer Outcomes
Source: JAMA Netw Open. 2024 Mar 20;7(3):e242976. doi: 10.1001/jamanetworkopen.2024.2976 (PMC10955379; doi:10.1001/jamanetworkopen.2024.2976)
Supplement: Supplement 1. — eFigure. Cohort Selection [file jamanetwopen-e242976-s001.pdf]

Supplemental Online Content

McKay RR, Nelson TJ, Pagadala MS, et al. Adrenal-permissive germline *HSD3B1* allele and prostate cancer outcomes in the Veterans Affairs health system. *JAMA Netw Open.* 2024;7(3):e242976. doi:10.1001/jamanetworkopen.2024.2976

eFigure. Cohort Selection

This supplemental material has been provided by the authors to give readers additional information about their work.

© 2024 McKay RR et al. *JAMA Network Open.*

eFigure. Cohort Selection

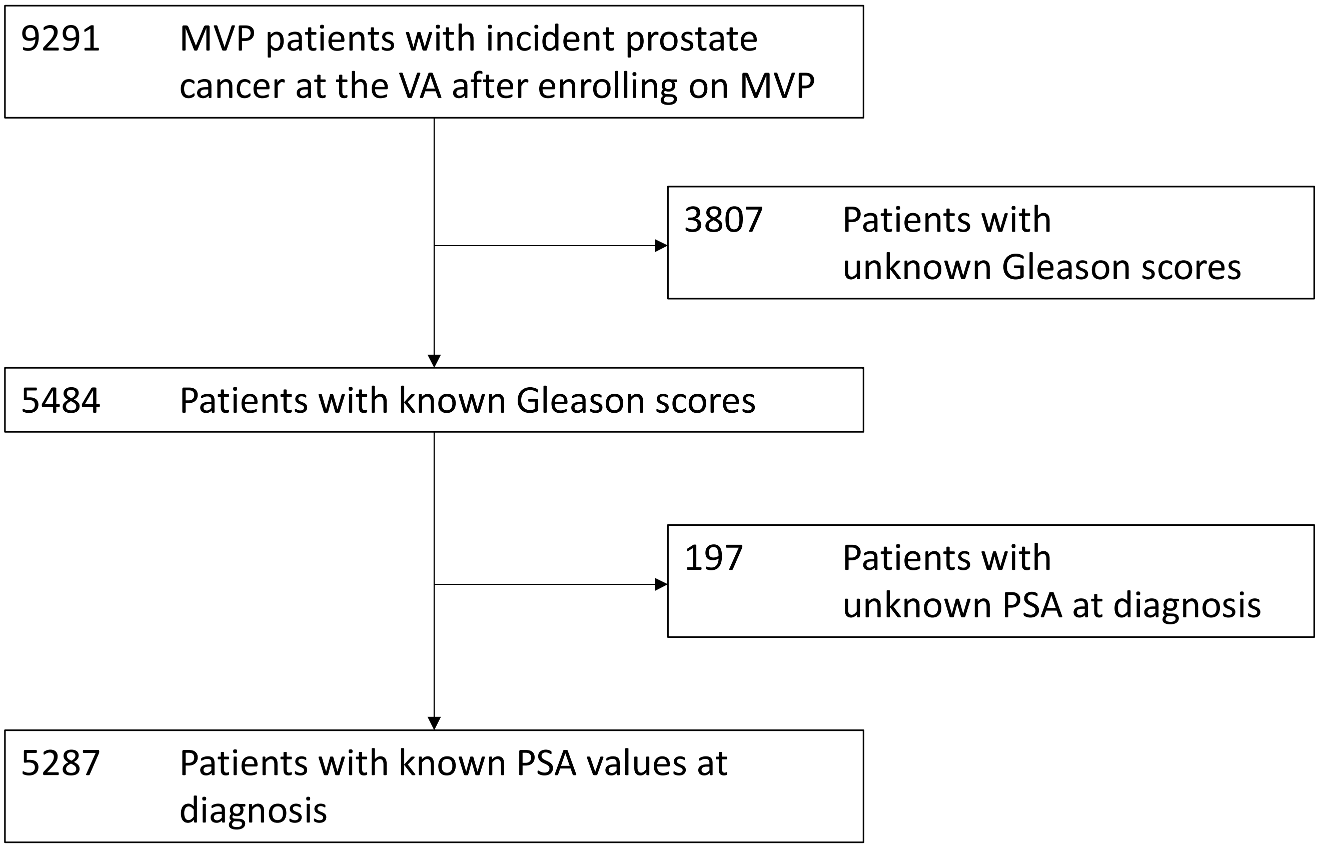

© 2024 McKay RR et al. *JAMA Network Open*.
