# Supplementary material for: Adrenal-Permissive Germline HSD3B1 Allele and Prostate Cancer Outcomes
Source: JAMA Netw Open. 2024 Mar 20;7(3):e242976. doi: 10.1001/jamanetworkopen.2024.2976 (PMC10955379; doi:10.1001/jamanetworkopen.2024.2976)
Supplement: Supplement 2. — Data Sharing Statement [file jamanetwopen-e242976-s002.pdf]

Data Sharing Statement

McKay. Adrenal-Permissive Germline HSD3B1 Allele and Prostate Cancer Outcomes in the Veterans Affairs Health System. *JAMA Netw Open*. Published March 20, 2024. doi:10.1001/jamanetworkopen.2024.2976

Data
Data available: Yes
Data types: Data dictionary, Deidentified participant data, Data (not involving human participants)
How to access data: Patient-level data are already accessible to all VA researchers with appropriate IRB approvals.
When available: With publication

Supporting Documents
Document types: None

Additional Information
Who can access the data: Patient-level data are currently accessible to all VA researchers with appropriate IRB approvals.
Types of analyses: Data is available for any type of analysis.
Mechanisms of data availability: Data will be made available after IRB approval.
